# Salicylic Acid in Aural Discharges

**Published:** 1876-04

**Authors:** Julian J. Chisolm

**Affiliations:** Professor of Eye and Ear Diseases in the University of Maryland, and Surgeon in charge of the Baltimore Eye and Ear Institute


### SALICYLIC ACID IN AURAL DISCHARGES.

By JULIAN J. CHISOLM, M. D., Professor of Eye and Ear Diseases in the University of Maryland, and Surgeon in charge of the Baltimore Eye and Ear Institute.

The advice usually given, to allow a running from the ear to continue, lest the sudden drying up of a long existing discharge should affect the lungs, or fall upon some other important organ, is as absurd as that the stopping of a leak in a barrel will injuriously affect the contained fluid, or must necessarily break out somewhere else. The reason for this wide-spread statement, in such constant use with the professional public, probably lies in the acknowledged difficulty in arresting aural discharges when they have become chronic. Much of this trouble is occasioned by want of cleanliness. In most cases, patients will keep the external parts of the meatus scrupulously clean, while the ulcerated drum surfaces are in a perpetual bath of decomposing, offensive and irritating pus. Remove frequently and thoroughly this irritating fluid, from the very bottom of the aural passage, and not only will all disagreeable odor cease, but a few of the cases of long-continued otorrhœa will undergo a positive cure. The majority of these cases will, however, require a more active treatment. No applications can be effective for aural discharges, if they are not brought into immediate contact with the raw surface from which the discharge oozes. Thorough cleansing must, therefore, precede all applications. The ear-drops in common use, viz : alum or zinc solutions, combined with carbolic acid, are very useful in many cases, but will not stop all discharges. In my own experience, I have found three or four remedies very efficacious in checking rebellous otorrhœa. One is a strong solution of nitrate of silver, from ten to sixty grains to the ounce of water. Another very useful application is an alcoholic solution of corrosive sublimate, two grains to the ounce. I have also used, with excellent effect, in very obstinate cases, chromic acid, five grains, to aquæ one ounce. These remedies are all efficient, but must be applied with care, by the surgeon himself, and be desisted from when they cause too much pain.

Seeing reports of the advantages derived from sprinkling powdered salicylic upon the surface of chronic ulcers, it was inferred that this new disinfectant may prove of value in aural surgery. My first trial was with the acid in aqueous solution. I found its solvent powers in water too weak for efficiency. Alcohol takes up the acid much more freely; an ounce of alcohol will dissolve a drachm of the acid, where a similar quantity of water would not take up two grains. Alcohol is, therefore, a good menstruum for an ear-drop containing salicylic acid. My experience was chiefly with the acid in powder, diluting it in varied proportion with calcined magnesia or oxide of zinc. I found that the pure salicylic acid could be very safely applied to the discharging tympanic membrane, without causing severe pain. The proportion which I found of most value, was one part of salicylic acid to two parts of magnesia powder, as a diluent. The ear being first thoroughly cleansed, the powder was blown into the ear through a quill, or by means of a laryngeal powder-puff. It should thoroughly cover the exposed secreting surfaces, from which the purulent discharge is constantly oozing. The effect in all cases has been good; its controlling influence being much more marked in some persons than in others. In some rare cases I have stopped an aural discharge by a single application. In most cases, it has to be repeated from day to day before this good result is secured.

My experience with salicylic acid has been so much more satisfactory than any other remedy heretofore tried, that I hasten to invite the body of the profession, who have these annoying cases to contend with, to add their experience to my own as to its usefulness in the arrest of chronic aural discharges. I will give but one case in illustration:

Mr. R., aged 37, of South Carolina, applied to the Baltimore Eye and Ear Institute for treatment, under the following circumstances: He had scarlet fever when a child, and for twenty-five years, at least, has had a constant discharge from the ear, which, up to the present time, he has, in vain, attempted to stop. His general health is excellent. Upon examination, I found the drum membrane destroyed, and the lining membrane of the drum cavity thick and red. The surface was at first concealed from view by a thick, yellow, offensive fluid, which filled the meatus. Having thoroughly cleansed the entire passage, I blew the mixed powder into the open drum cavity. Its presence caused only a sensation of warmth for a few moments. After twenty four hours the ear was again examined. The discharge was found in much diminished quantity, of a white color, and watery in consistency. The ear was thoroughly cleansed, and the powder reapplied. On the third day there was only fluid enough secreted in the twenty-four hours to moisten the powder, all odor having disappeared. The ear was cleansed anew and the powder again applied, in a very thin layer, upon the secreting mucous surface. On the fourth day the powder was found in the ear dry. From this time the secretion of twenty five years' standing seemed to be perfectly and permanently suppressed.

In the case of Mr. W., of Maryland, a constant discharge of twelve month's duration, had been with difficulty arrested by means of a strong solution of nitrate of silver. Three weeks afterward he exposed himself, in river bathing, when the aural discharge reappeared in full intensity. One thorough cleansing of the ear, with application of the mixed powder of salicylic acid and magnesia, at once arrested the discharge. At the next visit, after twenty-four hours, the ear was found perfectly dry, and it continued so.

I do not infer that all cases of aural discharge will be thus magically influenced by the new remedy, but such things have been done with it, and I feel satisfied that it will prove equally effective when judiciously used by others.—*Western Lancet.*

